# A naturally occurring polyphenolic compound for liver cancer treatment

**DOI:** 10.7150/ijms.127785

**Published:** 2026-10-02

**Authors:** Ming-Hui Yang, Yu-Chang Tyan, Hsin-Rou Liang, Chia-Yen Dai, Yung-Jen Chuang, Cheng-Hui Yuan, Shih-Chang Chuang

**Affiliations:** 1 Division of General and Digestive Surgery, Department of Surgery, Kaohsiung Medical University Hospital, Kaohsiung Medical University, Kaohsiung 807, Taiwan.; 2 Department of Medical Imaging and Radiological Sciences, Kaohsiung Medical University, Kaohsiung 807, Taiwan.; 3 Center of Teaching and Research, Kaohsiung Municipal Siaogang Hospital, Kaohsiung 812, Taiwan.; 4 Graduate Institute of Animal Vaccine Technology, College of Veterinary Medicine, National Pingtung University of Science and Technology, Pingtung 912, Taiwan.; 5 Department of Medical Research, Kaohsiung Medical University Hospital, Kaohsiung 807, Taiwan.; 6 Center for Tropical Medicine and Infectious Disease Research, Kaohsiung Medical University, Kaohsiung 807, Taiwan.; 7 Graduate Institute of Clinical Medicine, Kaohsiung Medical University, Kaohsiung 807, Taiwan.; 8 School of Medicine, College of Medicine, Kaohsiung Medical University, Kaohsiung 807, Taiwan.; 9 Division of Hepatobiliary and Pancreatic Medicine, Department of Internal Medicine, Kaohsiung Medical University Hospital, Kaohsiung Medical University, Kaohsiung 807, Taiwan.; 10 Department of Neurosurgery, E-Da Hospital, Kaohsiung 824, Taiwan.; 11 Mass Spectrometry Laboratory, Department of Chemistry, National University of Singapore, Singapore 119077, Singapore.

**Keywords:** hepatocellular carcinoma, liver cancer, piceatannol, polyphenolic compound

## Abstract

As a polyphenolic compound naturally found in nature, piceatannol is an analogue of resveratrol. These compounds share a common structural motif but have various substituents. Resveratrol shows anti-inflammation and anti-tumor properties, but such biological activities of the active resveratrol metabolite - piceatannol are unclear. In this study, the anti-tumor properties of piceatannol were investigated through cell viability, colony formation, migration assay, detection of reactive oxygen species (ROS) and mitochondrial membrane potential (MMP) change, reverse transcription quantitative polymerase chain reaction (RT-qPCR), and Western blotting in human hepatocellular carcinoma cell lines. Piceatannol decreased cell viability in a dose-dependent manner. These concentrations required to inhibit 50% of activity (IC_50_) for Huh7, HepG2, and Hep3B cells were 76.1-99.7, 17.0-19.6, and 57.0-83.7 μM after 24-72 hour treatment, respectively. Piceatannol inhibited the abilities of colony-forming and migration of HCC cells. Piceatannol may contribute to cell death in hepatocellular carcinoma cells, accompanied by altered MMP, reduced expression of HSPD1 and increased DNA double strand breaks. Our results show that piceatannol, the metabolite of resveratrol, supports an anti-cancerogenic effect by inhibiting proliferation and promoting cell death. Moreover, piceatannol may be a more potent anticancer agent than resveratrol. Hence, piceatannol could be a candidate for anti-cancer compound for the management of liver cancer.

## Introduction

As the most prevalent type of liver cancer, hepatocellular carcinoma (HCC) is a deadly disease, ranking as the third highest cause of cancer fatalities globally, accounting for more than 90% of primary liver cancers 1. This refers to a malignant tumor initiating from either hepatocytes or intrahepatic bile duct epithelial cells, and can be composed of hepatocellular carcinoma, cholangiocarcinoma or heterogeneous cancers. Liver cancer has been identified as a possible consequence of chronic liver disease and cirrhosis. The condition is often associated with factors like hepatitis B or C virus infection, excessive alcohol consumption, and nonalcoholic steatohepatitis 2. Despite considerable progress in the management of various malignancies, treatment options for liver cancer, a highly lethal malignancy, remain limited. Common treatments include liver resection, radiofrequency ablation, chemoembolization and liver transplantation. These methods are effective for HCC patients in early stages because they preserve normal liver function. However, many patients are not eligible for the above treatments since they are already in advanced stages of the disease when diagnosed. As a result, a high proportion of patients suffer from metastasis or recurrence. In addition, due to the resistance to chemotherapy or radiotherapy, the efficacy of alternative or palliative therapies is limited resulting in a 5-year survival of 18% 3. Thus, the burden of HCC is still high.

Treatments of targeted cancer therapy, immunotherapy and their combinations are crucial in advanced or unresectable HCC patients, but these only partially fulfill the medical intervention. For that reason, the development of new therapeutic strategies is still needed. One therapeutic option is traditional Chinese herbal medicine. Recent advancements have led to the processing of certain traditional Chinese medicine into standardized dosage forms, resulting in the development of Chinese patent drugs. Some of these were found to have the benefits of well-tolerated, and targeting of multiple factors/pathways and were applied in clinical trials 4. However, Chinese patent drugs (or Traditional Chinese Medicine, TCM) are primarily limited to oral administration, which is associated with low bioavailability. The nanonization of TCM and the development of nano-drug delivery systems offer promising strategies to overcome these hurdles by enhancing targeting specificity, improving bioavailability, and achieving sustained drug release. Nanotechnology-based strategies for TCM can be classified based on the use of carriers. Carrier-based systems include lipid carriers, polymeric carriers, inorganic carriers, and protein carriers, whereas carrier-free strategies encompass self-assembled herbal nanomaterials, TCM-derived carbon dots, and vesicle-like nanoparticles. Despite these advantages, nano-processing may alter the physicochemical interactions among TCM components, leading to uncertainty regarding the active constituents. Moreover, the majority of current nano-drug formulations remain at the laboratory stage and require further comprehensive safety and toxicological evaluation 5, 6. The other possible therapeutic option is to use natural bioactive compounds. Some compounds aim on changing the tumor immunosuppressive microenvironment 7. Other herbal products are capable of promoting apoptosis & necrosis, having characteristics of antioxidant, anti-inflammatory, antifibrotic, antiproliferative effect and antiviral activities in addition to inhibition of tumor metastasis 8, 9. These studies utilized both cell and animal models to evaluate the efficacy. Still, further studies are needed to explore their pharmacological mechanisms in addition to having clinical trials investigating efficacy and safety.

One of the most popular natural compounds is resveratrol. Resveratrol has demonstrated several defensive and protective effects including neuroprotective, antibacterial, anti-aging, cardioprotective, neuroprotective, vasorelaxant, estrogenic, anticancer, anti-inflammatory, and antioxidant effects 10, 11. However, the administration of resveratrol has a few drawbacks, including low aqueous solubility, low stability, and low bioavailability 10. It has been shown that resveratrol suppressed the proliferation of HCC cells with the half maximal inhibitory concentration (IC_50_) over 100 μM 12. In other words, it is essential to use high dosages of resveratrol to inhibit HCC cell proliferation, which may indicate the low bioavailability of resveratrol. Using derivatives of resveratrol may bypass the limitation. For example, pterostilbene is a methoxylated derivative of resveratrol and has greater bioavailability when administered orally 13. Nevertheless, the IC_50_ values of pterostilbine in various HCC cell lines were around 20-40 μM, which were relatively high 14. Piceatannol is a derivative of resveratrol having a significantly higher antioxidant capacity compared with that of resveratrol 15. Yet the antitumor effect of piceatannol against HCC remains vague and requires further investigation.

The present study aimed to investigate the anti-tumor effects of piceatannol against hepatocellular carcinoma. We revealed that piceatannol suppressed cell growth and migration in HCC cells, involving modulation of mitochondrial function and induction of cellular stress responses. These findings suggest that piceatannol represents a promising therapeutic strategy for HCC.

## Material and Methods

### Cell lines and reagents

The human hepatocellular carcinoma cell lines Huh7, HepG2, and Hep3B were kindly provided by Prof. Yu-Chang Tyan (Kaohsiung Medical University). Cells were maintained in DMEM (Dulbecco's Modified Eagle Medium, Gibco BRL, Grand Island, NY, USA) enriched with 10% fetal bovine serum (FBS, HyClone, Logan, UT, USA), penicillin (100 U/mL), streptomycin (100 μg/mL), and L-glutamine (2 millimolar) under a controlled incubator with 5% CO_2_. Piceatannol (#QF-7073) was purchased from Combi-Blocks (Nanjing, China). DMSO served as the vehicle control.

### Cell proliferation assay

Cell proliferation assay was performed to evaluate the effect of piceatannol on cell viabilities and was detected using an MTT proliferation assay. This assay was modified from the method of Yang et al. 16. Briefly, approximately 3 × 10³ cells per well were seeded into 96-well plates. After the incubation of 24 hours, cells were treated with 0, 20, 30, 100, 150 or 200 μM of piceatannol or resveratrol. For the comparison, cells were treated with 0, 10, 20, 30, 50 or 100 μM of sorafenib. Following drug exposure for the indicated treatment period (24-72 hours), MTT solution was added and allowed to incubate for 1.5 hours. Then, DMSO was added to each test well and absorbances were measured using a microplate reader (BioTek Synergy H1, Agilent, Santa Clara, CA, USA) at a wavelength of 540 nm. Cell viability was calculated as the percentage of absorbance relative to vehicle-treated controls. IC_50_ values were determined using a four-parameter logistic (4PL) regression model in GraphPad Prism. The dose-response data were analyzed by nonlinear regression, and the IC_50_ values were calculated from the fitted dose-response curves. Data were mean ± SD of three technical replicates. No inferential statistical tests were performed; results are presented descriptively.

### Colony formation assay

The effect of piceatannol on long-term proliferative capacity was assessed using a colony formation assay modified from Kant et al. 17. Briefly, cells were seeded in 6-well plates at a density of 3 × 10³ cells per well and allowed to attach for 24 h before treatment. Cells were subsequently treated with 0, 20 or 200 μM of piceatannol. The culture medium was changed every 2-3 days. After 14 days, cells were rinsed with PBS (phosphate-buffered saline) and fixed with 10% formaldehyde at room temperature. Colonies were stained with crystal violet at room temperature for 2 minutes before being photographed. For quantitative analysis, stained colonies were dissolved in 10% acetic acid and the absorbance of 595 nm was measured by a microplate reader (BioTek Synergy H1, Agilent, Santa Clara, CA, USA). Data were mean ± SD of three technical replicates. No inferential statistical tests were performed; results are presented descriptively.

### Wound healing assay

To assess the effect of piceatannol on cell migration, a wound healing assay from Yang et al. was adapted 18. Briefly, cells were seeded into 2-well culture inserts placed in 24-well plates and cultured until reaching confluence. After inserts removal, cells were treated with the indicated concentrations of piceatannol. Cell images were acquired at time points of 0, 24, 48 and 72 hours. Migration distances were measured and compared to that at the 0 hour. The wound healing process was monitored using an inverted microscope (Nikon Eclipse TS2, Tokyo, Japan) at 40× magnification. Images were acquired at the same physiological locations at 0 h and subsequent time points using Capture 2.2 software. The wound width was measured, and the migration rate was expressed as the percentage of wound closure, calculated using the following formula:



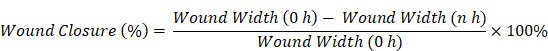



At least three randomly selected fields were analyzed per well, and the average value was used for statistical analysis.

### Reactive oxygen species (ROS) assay

Intracellular reactive oxygen species (ROS) production following piceatannol treatment was evaluated. Cells were treated with 0, 20, 30, 100, 150, or 200 μM piceatannol for 1, 2 or 24 h. Increased ROS may correlate with apoptosis or necrosis. The effect of piceatannol on the production of intracellular peroxide was assessed using a fluorescence microplate reader (BioTek Synergy H1, Agilent, Santa Clara, CA, USA) at excitation/emission wavelengths of 485/535 nm through a fluorometric probe (2,7-Dichlorodi-hydrofluorescein diacetate (DCFH-DA, CAYMAN# 20656). Cells were seeded at a density of 2 × 10^4^ cells per well and allowed to attach for eight hours before treating with piceatannol. Then, cells were grown under the medium containing DCFH-DA. After rinsing with PBS to remove excess probe, fluorescence signals were measured. Cells were incubated with 20 μM DCFH-DA for 45 min at 37 °C under dark conditions.

### Mitochondrial membrane potential assay

Cells were treated with 0, 20, 30, 100, 150, or 200 μM piceatannol for 2, 6, or 24 h. Normal mitochondrial function is essential for healthy cells. To measure the effect of piceatannol on the mitochondrial membrane potential, a mitochondria-specific cationic dye (5,5',6,6'-tetrachloro-1,1',3,3'-tetraethylbenzimidazolylcarbocyanine iodide, JC-1, AAT Bioquest # 22200) was used. Briefly, cells were seeded in 96-well plates (1x10^4^ cells per well) and then treated with a concentration gradient of piceatannol. Following the treatment, cells were incubated with JC-1 staining solution under dark conditions according to the manufacturer's instructions. Fluorescence was monitored using a fluorescent plate reader ((BioTek Synergy H1, Agilent, Santa Clara, CA, USA) at wavelengths of 475 nm (excitation)/530 nm (emission) and 535 nm (excitation)/590 nm (emission). Mitochondrial membrane potential alterations were measured through the ratio of fluorescence at 590 nm to 530 nm. A reduction in this red-to-green fluorescent intensity ratio was interpreted as mitochondrial depolarization. Data were mean ± SD of three technical replicates. No inferential statistical tests were performed; results are presented descriptively.

### Quantitative real-time polymerase chain reaction (qRT-PCR)

Cells were treated with 0, 20 or 200 μM of piceatannol for 48 h prior to RNA isolation. This assay was modified from the method of Livak and Schmittgen 9. Briefly, RNA from cells was prepared using TRIzol Reagent (Invitrogen, Carlsbad, CA, USA) and was reverse transcribed into complementary DNA (cDNA) using a Super Script II Reverse Transcriptase Kit (Invitrogen Inc., Carlsbad, CA, USA). Quantitative PCR was performed using KAPA SYBR FAST qPCR Master Mix reagent (Roche Diagnostics, Wilmington, MA, USA) on a QuantStudio 3 real-time PCR system (ThermoFisher, Waltham, MA, USA) to detect the relative mRNA expression levels of Heat shock protein family D member 1 (HSPD1) and TATA-box binding protein (TBP). Each reaction was performed in triplicate. The mRNA level was normalized using the TATA-box binding protein (TBP) mRNA level as the standard. The following primers were used: HSPD1 forward, 5′ ATGCTTCGGTTACCCACAGTC -3′ and reverse, 5′- AGCCCGAGTGAGATGAGGAG -3′; TBP forward, 5′-TGCACAGGAGCCAAGAGTGAA-3′ and reverse, 5′-CACATCACAGCTCCCCACCA-3′. Fold changes of gene expression were calculated as 2^-ΔΔCt^. Data were mean ± SD of three technical replicates. No inferential statistical tests were performed; results are presented descriptively.

### Western blotting

Cells were treated with 0, 20, 100, or 200 μM piceatannol for 6 and 24 h (for Caspase-3) and 48 h (for γH2AX) before protein extraction. Cells were lysed with radio-immunoprecipitation assay (RIPA) lysis buffer containing 50 mM Tris (pH= 7.5), 150 mM NaCl, 1% Triton X-100, 0.1% sodium dodecyl sulfate (SDS), and 0.5% sodium deoxycholate, This solution was enhanced by protease and phosphatase inhibitor cocktails (04693132001 and 04906837001, Sigma-Aldrich, St. Louis MO, USA). Total protein lysates were quantified using the Bradford assay (Bio-Rad#5000006). Approximately 30 μg total protein was loaded per lane, and equal amounts of proteins were separated by SDS-PAGE before transfer onto polyvinylidene fluoride (PVDF) membrane. Later, the PVDF membranes were blocked with 5% skim milk before incubating overnight at 4 °C with primary antibodies. Then, the membranes were rinsed with PBST and then incubated with diluted secondary antibodies (1:10000) at room temperature for two hours. After rinsing with PBST for a few times, the visualization of the protein band signals were achieved via Enhanced Chemiluminescent Reagent Kit (ECL) (Visual Protein; LF16-500) using Bio-Rad Western blot imaging systems (Bio-Rad, Hercules, USA). Band quantification was performed using Bio-Rad Image Lab (version 6.1) withα-Tubulin or GAPDH as the loading control. The following antibodies were used: Caspase-3 (1:2000, Proteintech; 19677-1-AP, Rosemont, IL, USA), γH2AX (1:1000, Sigma-Aldrich #SAB5600038, St. Louis, MO, USA), α-Tubulin (1:5000, Novus; NB100-690, Centennial, CO, USA) and GAPDH (1:1000, NB300-221, Novus Biologicals, Centennial, CO, USA).

### Statistical analysis

All collected data are expressed as mean ± standard deviation (SD). Statistical analyses were performed using GraphPad Prism 8.0 (GraphPad Software, San Diego, CA, USA). For multiple-group comparisons, one-way ANOVA was performed, followed by Dunnett's multiple comparison test to compare each treatment group with the control group. Statistical significance was defined as *p < 0.05, **p < 0.01 or ***p < 0.001.

## Results

### Piceatannol inhibits the proliferation of liver cancer cells

To analyze the cytotoxicity of piceatannol on cell proliferation, several liver cancer cell lines, including Huh7, HepG2, and Hep3B, were used. The cells were treated with increasing concentrations of piceatannol up to 72 hours. As shown in Figure 1, piceatannol showed effective antiproliferative activities of all tested liver cancer cells in a dose-dependent manner. The concentrations of piceatannol corresponding to 50% cell growth inhibition (IC₅₀) were determined to be 76.1-99.7 μM for Huh7 cells, 17.0-19.6 μM for HepG2 cells, and 57.0-83.7 μM for Hep3B cells after 24-72 hour treatment. Specifically, the IC_50_ values were determined as 94.1 μM for Huh7 cells, 17.4 μM for HepG2 cells, and 68.6 μM for Hep3B cells after 48 hour treatment. The IC_50_ values for resveratrol treatment were determined as 87.7 μM for Huh7 cells, 91.6 μM for HepG2 cells, and 171.5 μM for Hep3B cells after 48 hour treatment. The IC_50_ values for sorafenib treatment were determined as 4.3 μM for Huh7 cells, 5.4 μM for HepG2 cells, and 4.3 μM for Hep3B cells after 48 hour treatment. This indicates that all three cell lines are more susceptive to piceatannol compared with resveratrol where HepG2 cells are more sensitive to piceatannol compared with the other two cell lines.

### Piceatannol inhibits the colony formation capability of liver cancer cells

A clonogenic assay is an important method to assess the ability of adherent cells to survive, proliferate and form colonies. In order to evaluate the effect of piceatannol on the independent survival capacities of HCC cells, colony formation experiments were conducted with drug treatment of piceatannol (0, 20 or 200 μM) for 10-14 days, and colonies were stained by crystal violet to calculate the colony formation efficiency. The colony formation rate of the control group was set at 100 %. For Huh7 cells, the relative colony formation rates after piceatannol treatment were 84.8, and 22.1% at concentrations of 20 μM and 200 μM, respectively (Fig. 2A). For HepG2 cells, the relative colony formation rates for 20 μM and 200 μM piceatannol treatments were 11.2, and 8.4%, respectively (Fig. 2B). For Hep3B cells, the relative colony formation rates after piceatannol treatment were 8.5% and 7.1%, respectively (Fig. 2C). The results suggested that a substantial inhibition of the clonogenic survival capacity in tested HCC cells following the piceatannol treatment. Accordingly, the colony formation capabilities of HepG2 and Hep3B cells were appeared to decrease by piceatannol when compared with the control group. As for the groups of Huh7 cells, the inhibition indicated a dose-dependent manner (Fig. 2A).

### Piceatannol inhibits the migration capability of liver cancer cells

The enhanced migratory capabilities in HCC cells are linked to the poor prognosis 19. Hence, the effects of piceatannol on the migration of HCC cells were examined. Huh7, HepG2, and Hep3B cells were incubated with various concentrations of piceatannol (0, 20, 30, 100, 150 and 200 μM) for up to 72 h, and their migration capabilities were analyzed. The wound healing assay results showed that piceatannol treatment decreased the migration of HCC cells in a dose-dependent manner when compared with the untreated control groups (Figure 3). Additionally, in order to exclude the possibility that reduced migration was due to cytotoxicity, cell proliferation and migration under identical treatment conditions were simultaneously evaluated. Experimental conditions of cells treated with 20 μM piceatannol for 24 hours versus 0 μM piceatannol (control) for 24 hours were selected. MTT assay for Huh7 cells showed that the relative cell number increased by 8% compared to that of control, while relative migration ability declined from 27.07% to 16.61%, corresponding to a 38.6% suppression of migration in Huh7 cells. Similarly, MTT assay for Hep3B cells showed that the relative cell number decreased by merely 2% compared to that of control, whereas relative migration ability decreased from 9.6% to 5.8%, corresponding to a 39.6% suppression of migration in Hep3B cells. This indicates that cell viability remained essentially unaffected while migration ability was substantially inhibited by piceatannol at low dose. Our results indicated that piceatannol effectively weakened the migration capabilities of all tested HCC cells.

### Piceatannol affects the intracellular ROS levels of liver cancer cells

Reactive oxygen species (ROS) are chemically reactive molecules that play a pivotal role in regulating intracellular redox homeostasis. The onset of ROS production usually takes place within hours following cellular drug uptake, culminating in the induction of apoptosis within the subsequent 24-48 h. While basal levels of ROS are essential for normal cell signaling, excessive ROS accumulation is associated with oxidative stress, which has been implicated in the pathogenesis of numerous diseases, including cancer. Elevated ROS production has been closely linked to the activation of oncogenic signaling pathways that drive cellular proliferation, epithelial-to-mesenchymal transition (EMT), and cancer metastasis 20. Given these considerations, the ability of piceatannol to modulate intracellular ROS levels was evaluated across three HCC cell lines. Huh7, HepG2, and Hep3B cells were treated with various concentrations of piceatannol prior to measurement of intracellular ROS levels using fluorescent probes. As shown in Figure 4B, 4E and 4F, piceatannol appeared to increase intracellular ROS levels in HepG2 and Hep3B cells in the higher concentrations for 1 or 2 hour treatment group. A comparable increase in ROS levels was also observed in Huh7 cells under the same treatment conditions (Figure 4A and 4D). After 24 hour treatments, ROS levels in all three cell lines were reduced to varying degrees (Figure 4G-I).

### Piceatannol affects the mitochondrial membrane potential of liver cancer cells

Mitochondrial membrane potential (MMP) is a well-established indicator of mitochondrial integrity. Its loss is widely employed as a marker of apoptosis and typically manifests within 6 to 24 h post-treatment. Under normal physiological conditions, a stable MMP is essential for ATP synthesis and mitochondrial function; however, during apoptosis, dissipation of MMP reflects the permeabilization of the outer mitochondrial membrane and the subsequent release of pro-apoptotic factors such as cytochrome c. To evaluate whether cell death following piceatannol treatment involves mitochondrial dysfunction, MMP was assessed using the JC-1 fluorescent dye assay, in which a shift from red (J-aggregate, high MMP) to green (JC-1 monomer, low MMP) fluorescence indicates mitochondrial depolarization. As shown in Figure 5G-I, 24 hour treatment with piceatannol was associated with a marked loss of mitochondrial membrane integrity across all three tested cell lines, regardless of the concentration applied, suggesting that mitochondrial depolarization is a consistent downstream effect of piceatannol treatment in hepatocellular carcinoma cells. It is interesting to note that piceatannol treatment was associated with a trend toward reduced mitochondrial membrane integrity in Huh7 cells across the three tested time points, with similar trends observed across the concentrations tested (Figure 5A, D and G). These findings suggest a more sensitive mitochondrial priming threshold in Huh7 cells.

### Piceatannol affects the mRNA expression of heat shock protein family D member 1 (HSPD1) of liver cancer cells

Heat shock protein family D member 1 (HSPD1) is crucial to mitochondria because imported proteins require HSPD1 for folding and assembly. Besides, HSPD1 may stabilize mitochondrial survivin and blocks programmed cell death in a tumor, indicating its tumorigenic role 21. Figure 6 shows the quantified mRNA expression levels of HSPD1 in cells after treatment with 0, 20 or 200 μM of piceatannol. The results indicated that increasing concentrations of piceatannol were associated with progressively reduced HSPD1 expression levels.

### Piceatannol affects the double-strand breaks of liver cancer cells and may contribute to differential protein expression of total and cleaved Caspase-3

Histone H2AX can be rapidly phosphorylated following exposure to substances associated with DNA damage. Thus, gamma-H2AX can quantitatively reflect DNA damage associated with the treatment, show dose-dependent results and can be a widely used biomarker. As shown in Figure 7, γ-H2AX expression levels vary among cells exposed with 0, 100, and 200 μM piceatannol. When piceatannol doses increased, the expression levels of γ-H2AX increased as well.

Besides, in order to evaluate the pro-apoptotic effects of piceatannol, Caspase-3 activation across three distinct cell lines were analyzed. As shown in Figure 8, no appreciable Caspase-3 activation was observed in Huh7 cells at either time point, indicating that Huh7 cells are resistant to cell death following piceatannol treatment at this concentration and fail to initiate the apoptotic cascade, as evidenced by the absence of cleaved Caspase-3 upregulation. This is consistent with the MTT results, which showed an approximately 8% increase in relative cell number following 20 μM piceatannol treatment for 24 hours compared to the untreated control. Regarding HepG2 cells, an apoptotic response was observed following treatment, with cleaved Caspase-3 levels showing a non-significant increase at 6 h and a significant increase at 24 h. This persistent activation of the executioner Caspase is in agreement with the reduction in cell viability. Specifically, the MTT assay showed a profound effect, with the relative cell number plummeting from 100% in the control group to 49% after 24 h of treatment. In Hep3B cells, a distinct dynamic pattern emerged. Cleaved Caspase-3 protein levels showed a marked increase at 6 h, whereas only a modest increase was observed at 24 h, with neither reaching statistical significance. This pattern may suggest a transient rather than sustained apoptotic signal, although this interpretation should be considered preliminary given the lack of statistical significance. In contrast, total caspase-3 levels showed a modest increase at 6 h and a more marked increase at 24 h, neither reaching statistical significance. This may indicate that between 6 and 24 h, a substantial amount of newly synthesized Procaspase-3 accumulated but was not subsequently processed into its active form. Thus, at the 24 h mark, the cells appeared to be primed for cell death yet arrested or suppressed, possibly through the engagement of compensatory pro-survival mechanisms. This observation is in close agreement with the MTT data, which showed only a marginal 2% reduction in relative cell number following 20 μM piceatannol treatment for 24 hours.

## Discussion

A malignant tumor which originates from hepatocytes or intrahepatic bile duct epithelial cells is the primary indicator of liver cancer 22. Due to the difficulties of early detection, limits of treatment options, and high recurrence rates, the incidence rate is approximately equal to the mortality. Without proper treatment, patients may expire within one year from the diagnosis. According to GLOBOCAN estimates, HCC accounts for approximately 10% of cancer-related deaths among males and 5% among females worldwide 23. Although the incidence of hepatocellular carcinoma is ascending along with the mortality rate, the new drug development is relatively slow. After the usage of sorafenib, regorafenib and lenvatinib in the treatment of hepatocellular carcinoma for years, the monoclonal antibody treatment using ramucirumab, nivolumab, or pembrolizumab has been permitted until recent years 24-28. Even though the systemic treatment has been improved, the treatment for unresectable tumors remains a challenge.

When probing for therapy, resveratrol, a phytochemical, has surfaced. Resveratrol (3,5,4′-trihydroxy-trans-stilbene) is a polyphenol (by structure) and a phytoalexin (by function) and can be isolated from plants. The *trans*-stereoisomer of resveratrol is the more active, abundant and stable form in various edible plants. Resveratrol has been tested against various types of cancer including HCC. The values of IC_50_ of resveratrol against Huh7, HepG2 and Hep3B cells were between 99.7 and 170.0 μM. In comparison, the IC_50_ of piceatannol against HCC cells were lower. Based on the results, it can be inferred that piceatannol exhibits a more pronounced inhibitory effect on cell proliferation compared to resveratrol. Interestingly, although resveratrol inhibited the ability of HepG2 and Hep3B cells to form colonies in a dose-dependent manner, our data showed that the ability of these two type cells to form colonies was inhibited by piceatannol at 20 μM, a relatively low concentration. The dose-dependent manner only manifested in Huh7 cells. Thus, piceatannol may not be highly cytotoxic for these cells, but it has the ability to inhibit long-term reproductive viability, which may prevent tumor regrowth.

Nevertheless, translating these in vitro results into clinical practice presents a substantial challenge, as the effective concentrations of piceatannol exceed currently achievable systemic plasma levels; thus, direct clinical translation in its current form is not feasible. However, precedent from structurally related polyphenols, including curcumin and resveratrol, demonstrates that nanoparticle-based delivery systems and structural modifications can substantially improve bioavailability. This offers a feasible translational pathway to bridge the gap between our in vitro findings and meaningful in vivo efficacy. For example, curcumin exhibits anticancer activity in vitro at concentrations of approximately 5-109 µM, yet oral administration yields free plasma levels far below these effective concentrations. To overcome this limitation, various nanoformulation strategies have been developed 29. In a randomized cross-over trial in healthy adults, micellar curcumin and a curcumin-γ-cyclodextrin complex achieved 57-fold and 30-fold increases in the area under the plasma concentration-time curve, respectively, compared to native curcumin 30. Similarly, resveratrol is commonly used at 10-200 µM in vitro, concentrations that far exceed the plasma levels attainable after a 1 g oral dose in humans, with an oral bioavailability of less than 1%. A self-emulsifying resveratrol-phospholipid complex has been shown to enhance bioavailability by up to 48-fold in a rat model 31. Notably, analogous formulation strategies have already been explored directly for piceatannol. Dhanapal and Ravindrran (2018) demonstrated that chitosan/poly-lactic acid-coated piceatannol nanoparticles (CS/PLA-PicNPs) exhibited enhanced cytotoxic activity against HepG2 cells, with an IC₅₀ of 5 µg/mL, compared with piceatannol alone, which yielded an IC₅₀ of 20 µg/mL 32. These precedents indicate that employing analogous formulation strategies may bridge the gap, rendering current piceatannol concentrations clinically viable for future applications.

Manipulating the amounts of reactive oxygen species (ROS) in hepatocellular carcinoma cells may have an anticancer effect. For resveratrol, it has been shown that resveratrol increased ROS production in several human hepatoma cell lines (C3A, SMCC7721 and HepG2 cells) 33, 34. Exceptionally, studies carried out on cell lines of leukemia, B16F10 melanoma, and rat hepatoma all showed the suppression of ROS generation by piceatannol 35-37. Interestingly, piceatannol treatment elicited a biphasic ROS response, characterized by an early transient surge followed by a substantial reduction after 24 hours. The initial ROS elevation likely reflects direct mitochondrial perturbation, consistent with the collapse of mitochondrial membrane potential (ΔΨm) observed at 6 hours. The subsequent ROS attenuation at 24 hours, which likely stems from a compensatory antioxidant response, may serve to decrease invasive activity.

Mitochondria are essential for cellular physiology; however, their dysfunction may contribute to deleterious effects on cell viability and function. Thus, cancer cells embrace stress response pathways to alleviate exogenous or endogenous cellular stresses and to improve their rates of survival and proliferation. One of the responsive pathways is the mitochondrial unfolded protein response (UPRmt), which is essential for maintaining protein homeostasis in mitochondria under stress conditions 38. Several chaperone proteins are involved in UPRmt in which HSPD1, a prominent marker associated with the UPRmt activation process, can be upregulated to restore mitochondrial proteostasis, thereby promoting cellular viability 39. Our data showed that piceatannol correlates with the mitochondrial potential disruption and reduced the mRNA levels of HSPD1. In Uçar's study, either resveratrol treatment or HSPD1 knockdown decreased the viability of glioblastoma cells 40. In addition, it has been shown that resveratrol treatment induced the disruption of mitochondrial membrane potentials in HCC cells 41. Similarly, piceatannol treatment also disrupted the mitochondrial membrane potentials in pancreatic cancer and neuroblastoma cells 42, 43. Thus, the downregulation of HSPD1 alongside mitochondrial damage suggests that piceatannol-related cytotoxicity is associated with a compromise in mitochondrial proteostasis. Furthermore, the evaluation of γH2AX protein expression revealed that piceatannol may contribute to a dose-dependent increase in its levels within HCC cells. While H2AX phosphorylation was clearly observed post-treatment, it is important to note that γH2AX is not a specific marker for oxidative DNA damage, as it may also arise from replication stress or apoptotic DNA fragmentation. Due to the lack of complementary assays, such as the comet assay, or mechanistic investigations to differentiate these possibilities, the precise mechanism driving γH2AX induction in response to piceatannol remains to be fully elucidated.

The absence of Caspase-3 activation in Huh7 cells following piceatannol treatment does not indicate its ineffectiveness but suggests that cell death may proceed through a non-Caspase-3-dependent pathway. Huh7 cells harbor the TP53 Y220C mutation, which produces a structurally destabilized p53 protein with impaired transcriptional activity. Frank et al. reported that mutant p53 proteins, including structural mutants, can bind Procaspase-3 at mitochondria and interfere with its Caspase-9-mediated activation 44. The potential interaction between Y220C mutant p53 and procaspase-3 remains speculative as it was not directly evaluated here. Our limited temporal resolution, together with the undetectable levels of cleaved caspase-3 in Huh7 cells, leaves open the possibility that the apoptotic response is either entirely absent or simply delayed. Additional time points and direct interaction assays (such as co-immunoprecipitation) are required in future studies to fully elucidate this proposed mechanism. Consistent with our observations, Rodríguez-Hernández et al. reported that sorafenib (10 µM) triggered Caspase-3 activation specifically in HepG2 and Hep3B, whereas it failed to induce a significant apoptotic response in Huh7 cells at this low concentration 45. Even at 100 µM, where Caspase-3 activity was significantly elevated in all three cell lines, the magnitude of activation in HepG2 and Hep3B remained approximately 2.5-fold and 2.1-fold higher than in Huh7, respectively; a similar pattern was observed with regorafenib (100 µM), which induced Caspase-3 activity at 2.6-fold and 1.7-fold higher levels in HepG2 and Hep3B compared to Huh7, respectively 45.

Furthermore, changes in mitochondrial membrane potential (MMP) were observed exclusively in Huh7 cells at 2 h. By 6 h, MMP reductions were observed in both Huh7 and Hep3B cells, with a concentration-dependent pattern in which greater depolarization was observed at higher piceatannol concentrations. At 24 h, MMP reductions were observed in all three cell lines (Huh7, HepG2, and Hep3B); however, this concentration-dependent pattern was no longer evident. When comparing the basal mitochondrial membrane potential (ΔΨm) among the three untreated cell lines, Huh7 cells exhibited a markedly hyperpolarized state relative to the others. When normalizing the basal ΔΨm value of Hep3B cells to 1.00, the basal ΔΨm values for Huh7 and HepG2 cells were 5.69 and 1.07, respectively, indicating that Huh7 cells harbors a substantially elevated basal mitochondrial membrane potential.

Based on the concentration-dependent, biphasic effect of piceatannol observed in Huh7 cells (pro-survival response at lower concentrations in contrast to severe mitochondrial impairment and cell death at higher concentrations), we propose that piceatannol exerts its anti-tumor efficacy through mitochondrial modulation. Our findings of mitochondrial membrane depolarization, altered Bcl-2/Bax balance (Supplementary Figure S1), and modulation of redox homeostasis support the involvement of multi-target effects on mitochondrial function. Consistent with this concept, He et al. (2024) demonstrated that Schisandrin A selectively exerts cytotoxicity against Huh7 cells via mitochondrial disruption, manifesting as dose-dependent cytotoxicity and near-complete suppression of clonogenic capacity at higher concentrations 46. Similar to Schisandrin A, piceatannol treatment was associated with concentration-dependent mitochondrial effects, suggesting that targeting mitochondrial function may represent a viable therapeutic strategy for hepatocellular carcinoma.

In our study, low-dose piceatannol treatment may similarly contribute to increased levels of cleaved Caspase-3 in HepG2 and Hep3B cells but not in Huh7 cells, mirroring the differential Caspase-3 activation pattern reported for sorafenib and regorafenib. We speculate that cleaved Caspase-3 accumulation in Huh7 cells may only become detectable at higher piceatannol concentrations; however, both our MTT data and prior reports indicate that high-dose treatment may contribute to extremely low numbers of viable cells, posing a technical challenge for protein detection. Therefore, direct assessment of Caspase-3 activation in Huh7 under high-dose piceatannol treatment was not feasible due to the limited number of surviving cells available for analysis. These results underscore that the blunted Caspase-3 activation in Huh7 cells stems from an inherent biological barrier, likely involving the sequestration of Procaspase-3 by mutant p53. Thus, the resistance observed is not a reflection of piceatannol's ineffectiveness, but rather suggests that Huh7 cells may rely on non-canonical cell death mechanisms. For example, LC3-II levels increased more than that of LC3-I in Huh7 cells following piceatannol treatment, whereas this pattern was not observed in HepG2 or Hep3B cells (Supplementary Figure S2). This finding correlates with distinct cellular responses in Huh7 cells at lower piceatannol concentrations, including a slight increase in cell viability, attenuated ROS accumulation, and suppressed Caspase-3 activation. However, LC3-II accumulation alone cannot distinguish between increased autophagy induction and impaired autophagic flux. One possible interpretation is that mild cellular stress correlated with piceatannol may contribute to autophagy-related responses preferentially in Huh7 cells, potentially contributing to the relative resistance of this cell line to Caspase-3-dependent cell death compared to HepG2 and Hep3B cells. Alternatively, LC3-II accumulation may reflect impaired autophagic flux secondary to mitochondrial dysfunction. As the present study did not include p62/SQSTM1 analysis or autophagy modulation experiments, the functional role of LC3-II accumulation and its contribution to the cell line-specific responses remain uncertain. Future studies incorporating these approaches will be necessary to clarify whether autophagy serves a cytoprotective or pathological role in piceatannol-treated Huh7 cells. Although cleaved PARP1 was detected in Huh7 cells following piceatannol treatment (Supplementary Figure S3), the absence of detectable cleaved caspase-3 remains a limitation of the present study and warrants cautious interpretation, as these findings alone do not establish classical caspase-3-dependent apoptosis. Consistent with these findings, Hoechst/PI staining revealed nuclear morphological changes consistent with apoptosis in all three HCC cell lines (Supplementary Figure S4), providing complementary morphological evidence.

A limitation of the present study is that the experiments presented in Figures 3, 4, 7 and 8 were based on independent biological replicates, whereas those in Figures 1, 2, 5, and 6 primarily involved technical replicates; consequently, the statistical interpretation of these findings differs in their ability to capture biological variability and support independent biological inference. Although these experiments provide supportive evidence for the observed treatment-related effects, the limited biological replication in Figures 1, 2, 5, and 6 may not fully capture biological variability, and mechanistic validation (e.g. rescue experiments or direct biochemical or DNA-damage assays) was not performed. Therefore, the associations reported herein should be interpreted as correlative and hypothesis-generating rather than mechanistically definitive, and further studies with additional independent biological replicates and mechanistic validation are needed to confirm the reproducibility, robustness, and causal basis of these findings. Despite these mechanistic uncertainties, the favorable safety profile of piceatannol supports its continued investigation as a potential therapeutic agent for HCC. In normal cells (human hepatic stellate cells) and an aged mouse (11.5 month-old), piceatannol suggested low toxicity (IC_50_ values indicating minimal cytotoxicity) and did not show signs of hepatotoxicity (Supplementary Tables S1 and S2). These findings, combined with its indicated anti-tumor activity, suggest that piceatannol, as a metabolite of resveratrol, may represent a more potent and safer anticancer agent than its parent compound.

## Conclusion

In conclusion, the present study suggests that piceatannol exerts concentration- and cell line-dependent anti-tumor activity against hepatocellular carcinoma cells. Piceatannol treatment was associated with suppressed cell proliferation, inhibited colony formation, impaired migratory capacity, altered intracellular ROS levels, downregulated HSPD1 expression, mitochondrial membrane depolarization, and increased markers of DNA double-strand breaks in Huh7 and HepG2 cells; a similar trend was observed in Hep3B cells but did not reach statistical significance. These findings provide a preliminary, hypothesis-generating foundation for the further investigation of piceatannol as a potential candidate for the management of hepatocellular carcinoma.

## Supplementary Material

Supplementary methods, figures and tables.

## Figures and Tables

**Figure 1:**
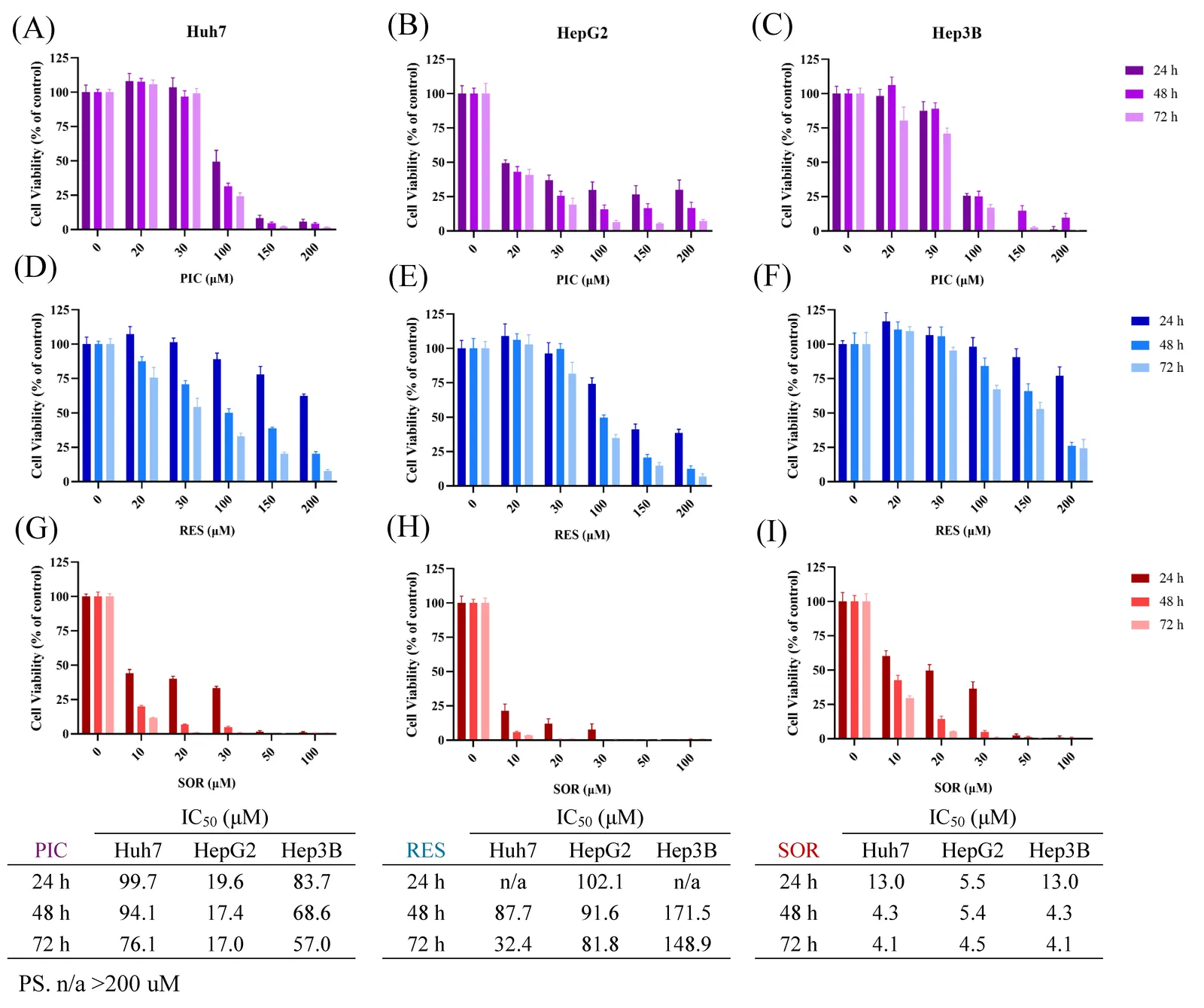
Piceatannol and resveratrol inhibited the proliferation of HCC cells differently. Huh7 (A), HepG2 (B), and Hep3B (C) cells were treated with 0, 20, 30, 100, 150 or 200 μM of piceatannol (PIC) at 24, 48, and 72 hours. Huh7 (D), HepG2 (E), and Hep3B (F) cells were treated 0, 20, 30, 100, 150 or 200 μM of resveratrol (RES) at 24, 48, and 72 hours. Huh7 (G), HepG2 (H), and Hep3B (I) cells were treated with 0, 10, 20, 30, 50 or 100 μM of sorafenib (SOR) at 24, 48, and 72 hours. Cell viabilities were determined by MTT assay. The percentages of viable cells normalized to the solvent-treated control were plotted. Data were mean ± SD of three technical replicates. No inferential statistical tests were performed.

**Figure 2:**
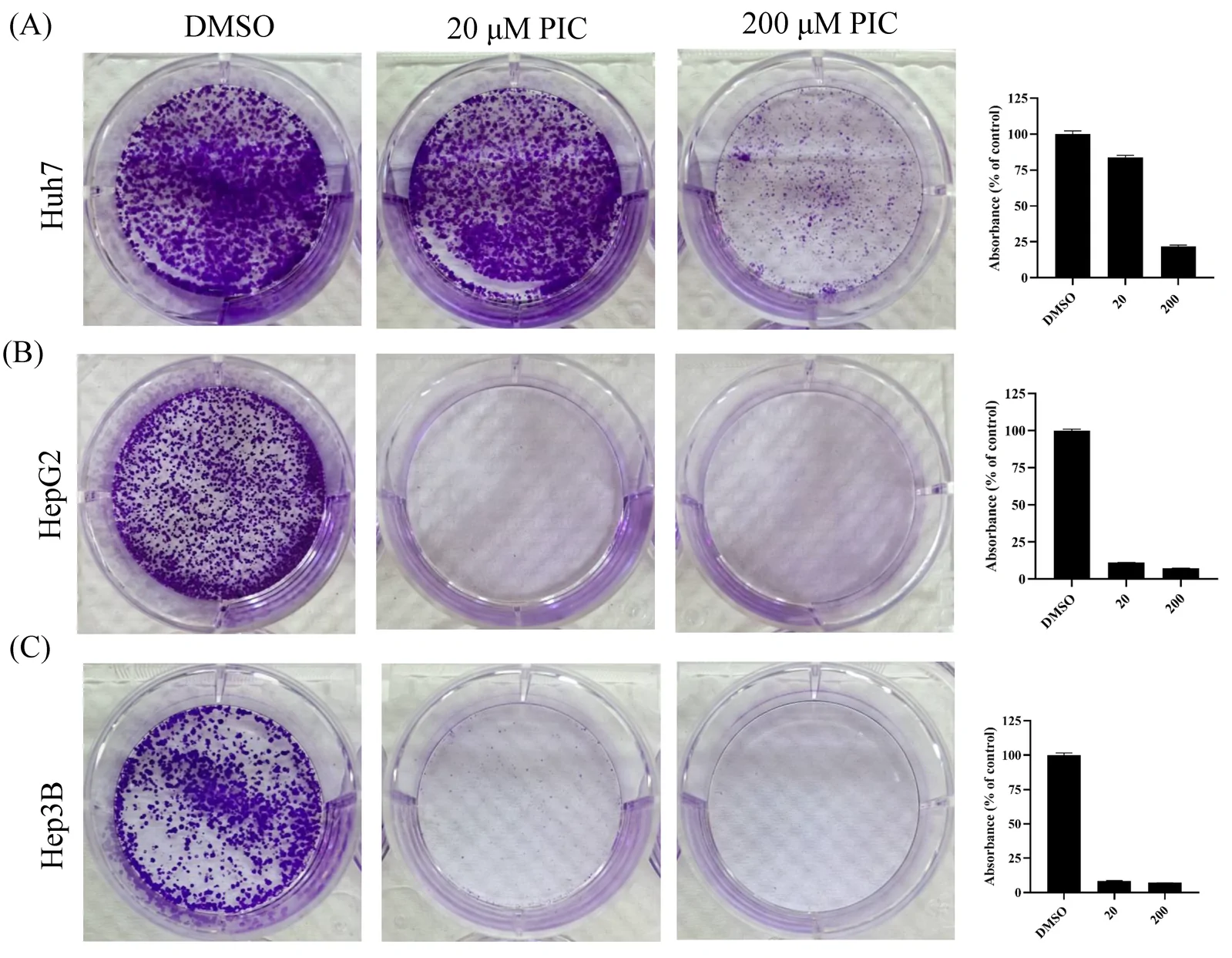
Piceatannol suppressed the colony formation capabilities of HCC cells. The colonies formed under the influence of solvent, 20 or 200 μM of piceatannol (PIC) were stained by crystal violet solution. Assays were performed in Huh7 (A), HepG2 (B) and Hep3B cells (C). Data were mean ± SD of three technical replicates. No inferential statistical tests were performed.

**Figure 3:**
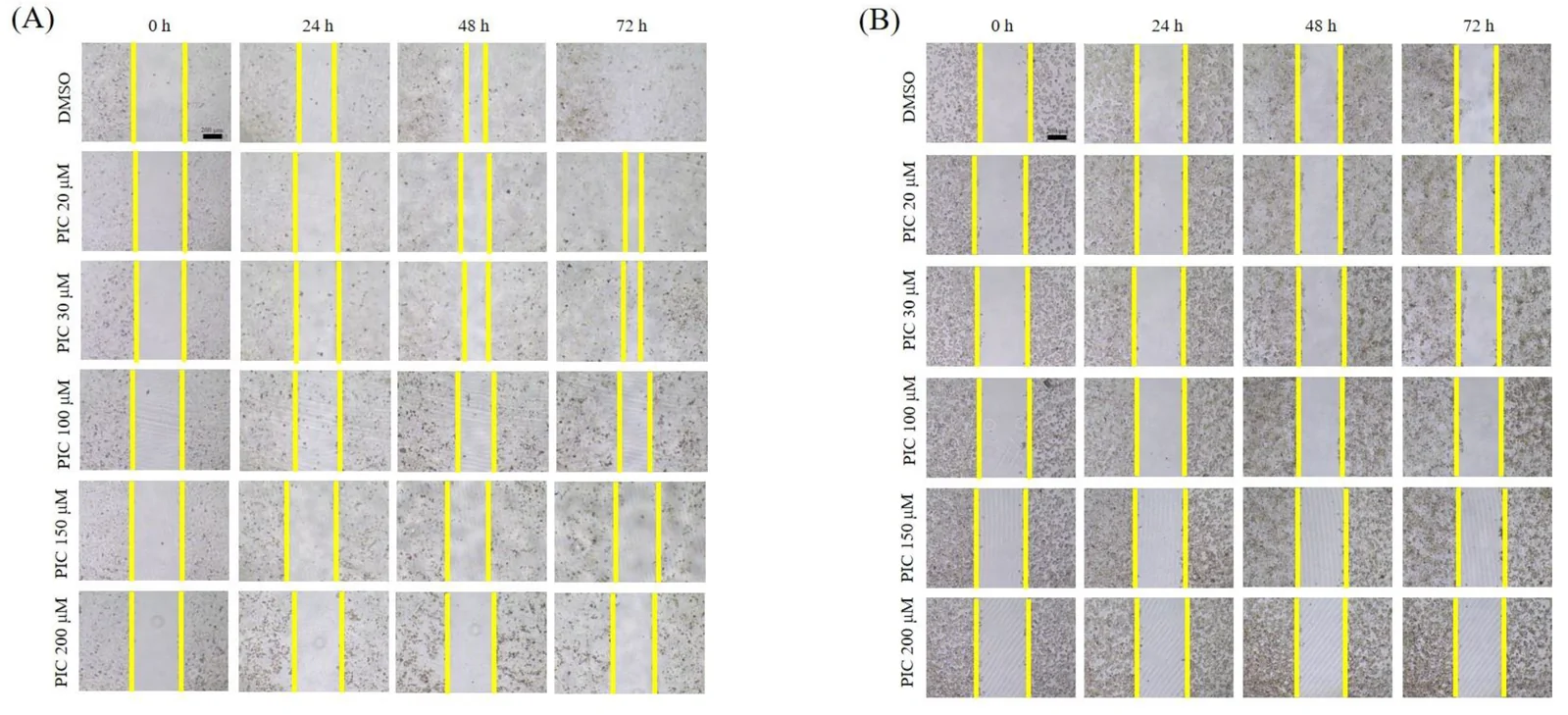

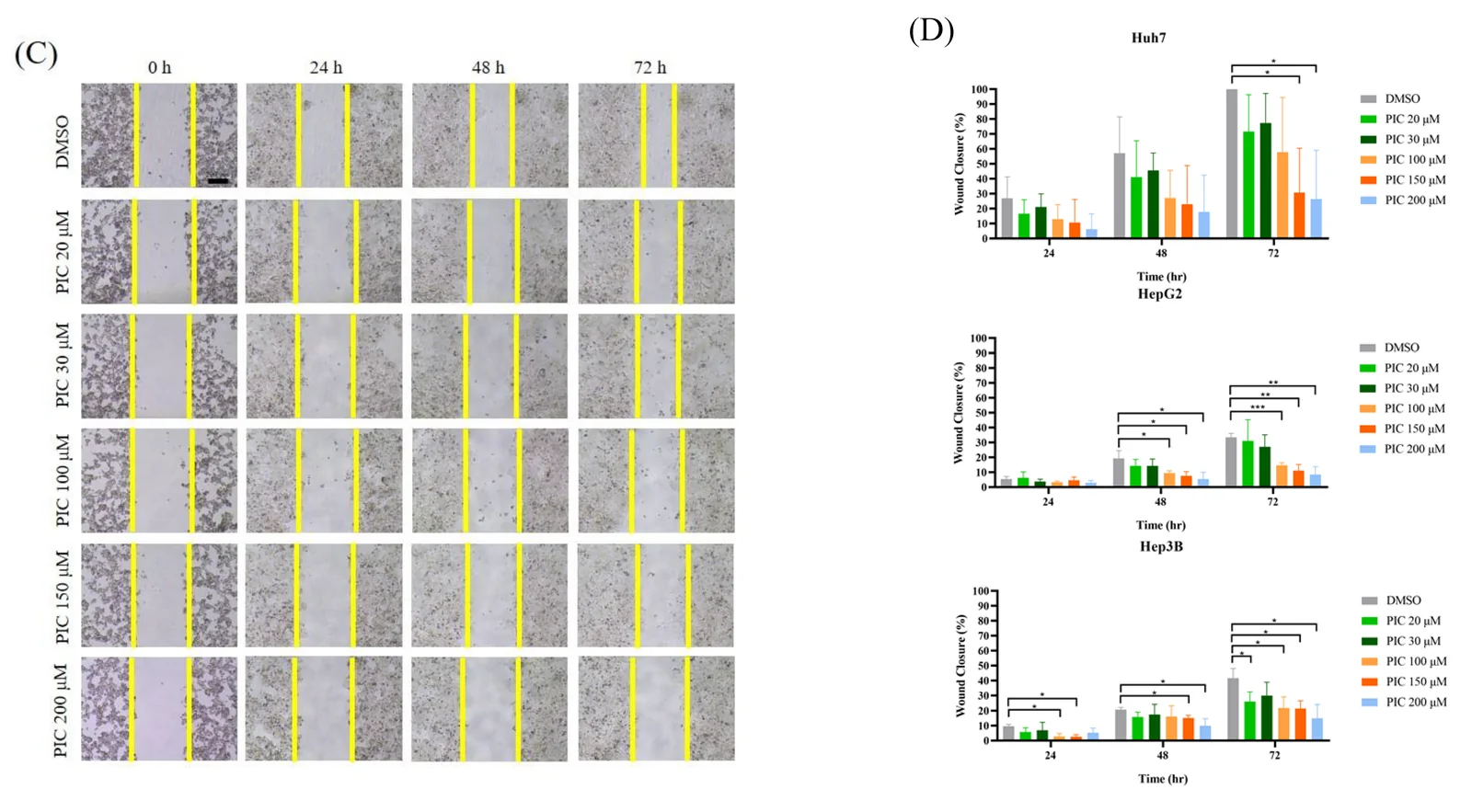
Piceatannol inhibited the migration of HCC cells. To evaluate the effect of piceatannol on cell migration, cells were seeded in the 2-well culture inserts. After reaching confluence, inserts were removed and cells were treated with various concentrations of piceatannol (PIC). The changes of the migration distances were detected by photographing at time points of 0, 24, 48 and 72 h. Assays were performed in Huh7 (A), HepG2 (B), and Hep3B cells (C). The quantitative analyses were also shown (D).Migration distances were measured and compared to that at the 0 hour time point. Results were presented as means ± the standard deviation (n = 3 biological replicates per group). Statistical significance was determined by one-way ANOVA followed by Dunnett's multiple-comparison test. *p < 0.05, ** p < 0.01, ***p<0.001 versus the control group.

**Figure 4:**
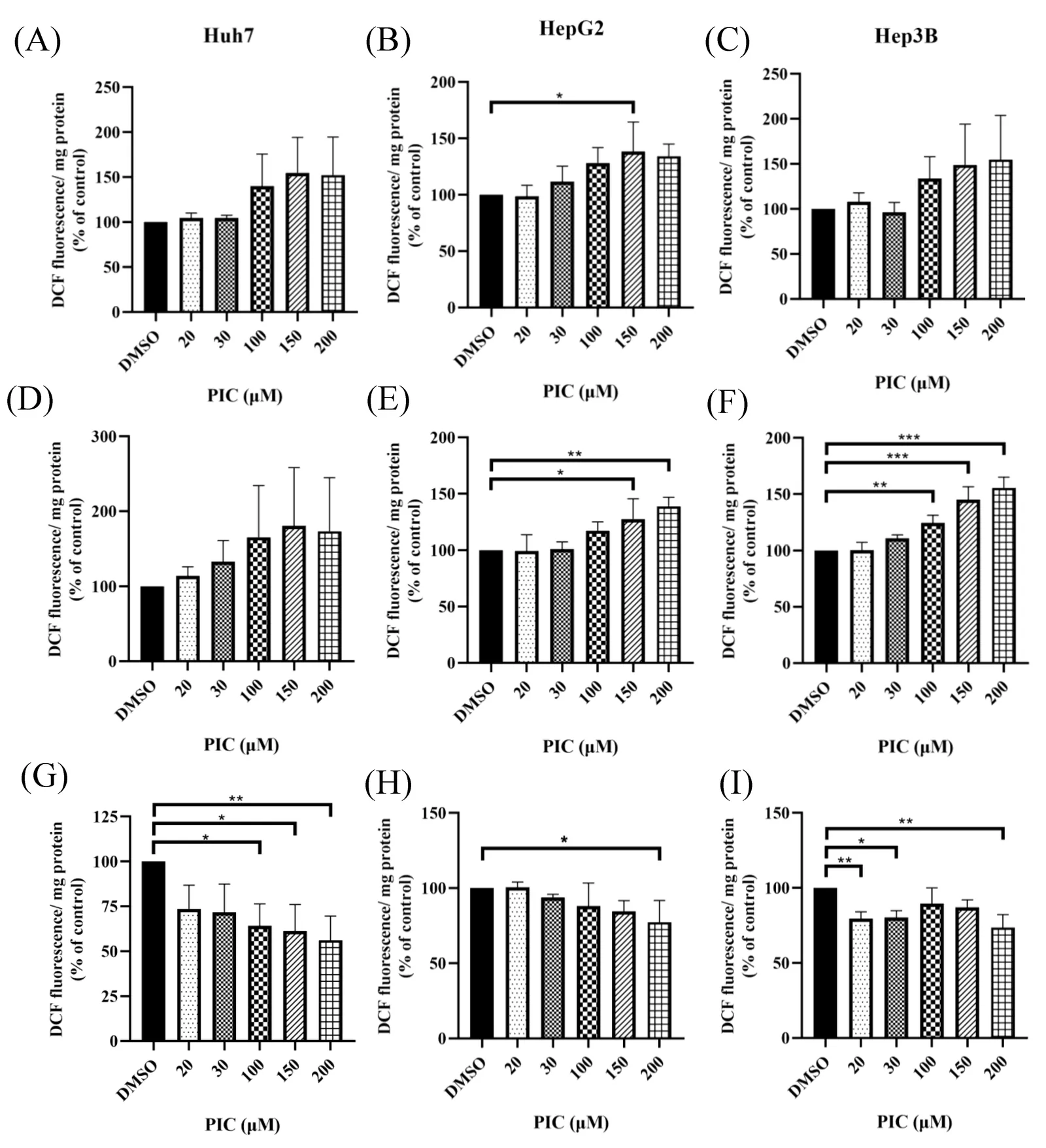
Piceatannol affected the intracellular ROS levels in HCC cells. The effect of piceatannol on the levels of intracellular ROS after cells treated with piceatannol (PIC) were detected and quantified using DCFH-DA. Huh7 (A, D, G), HepG2 (B, E, H), and Hep3B (C, F, I) cells were treated with 0, 20, 30, 100, 150 or 200 μM of piceatannol (PIC) for 1 h (A-C), 2 h (D-F), or 24 h (G-I). Data were expressed as mean ± SD (n = 3, biological replicates per group). Statistical significance was determined by one-way ANOVA followed by Dunnett's multiple-comparison test. *p < 0.05, ** p < 0.01, ***p<0.001 versus the control group.

**Figure 5:**
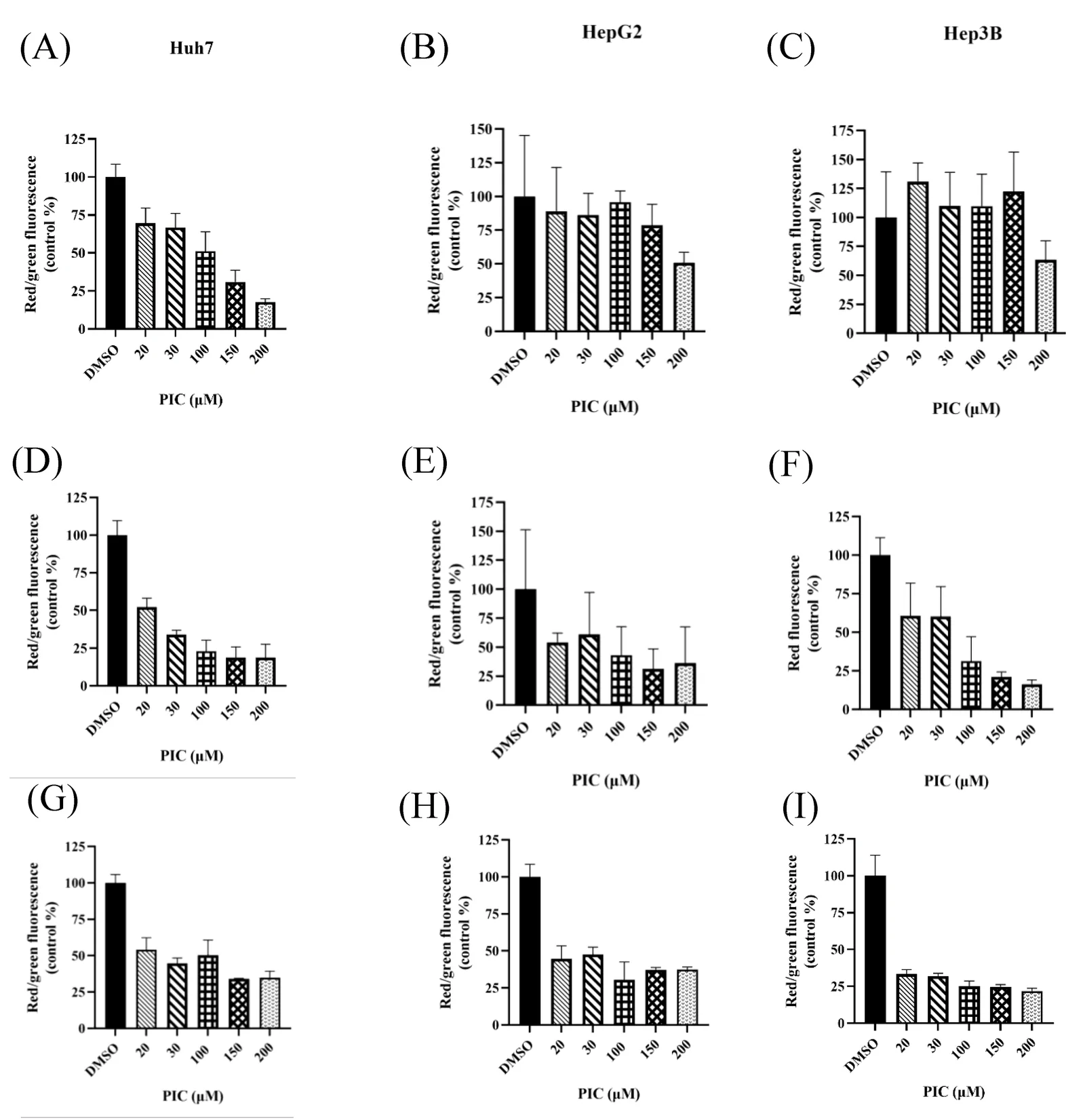
Piceatannol affected the mitochondrial membrane potentials in HCC cells. The changes of mitochondrial membrane potential after cells treated with piceatannol (PIC) were detected using JC-1. Huh7 (A, D, G), HepG2 (B, E, H), and Hep3B (C, F, I) cells were treated with 0, 20, 30, 100, 150 or 200 μM of piceatannol (PIC) for 2 h (A-C), 6 h (D-F), or 24 h (G-I). Data were mean ± SD (n = 6 for 2 and 6 h; n = 3 for 24 h, technical replicates per group). No inferential statistical tests were performed.

**Figure 6:**
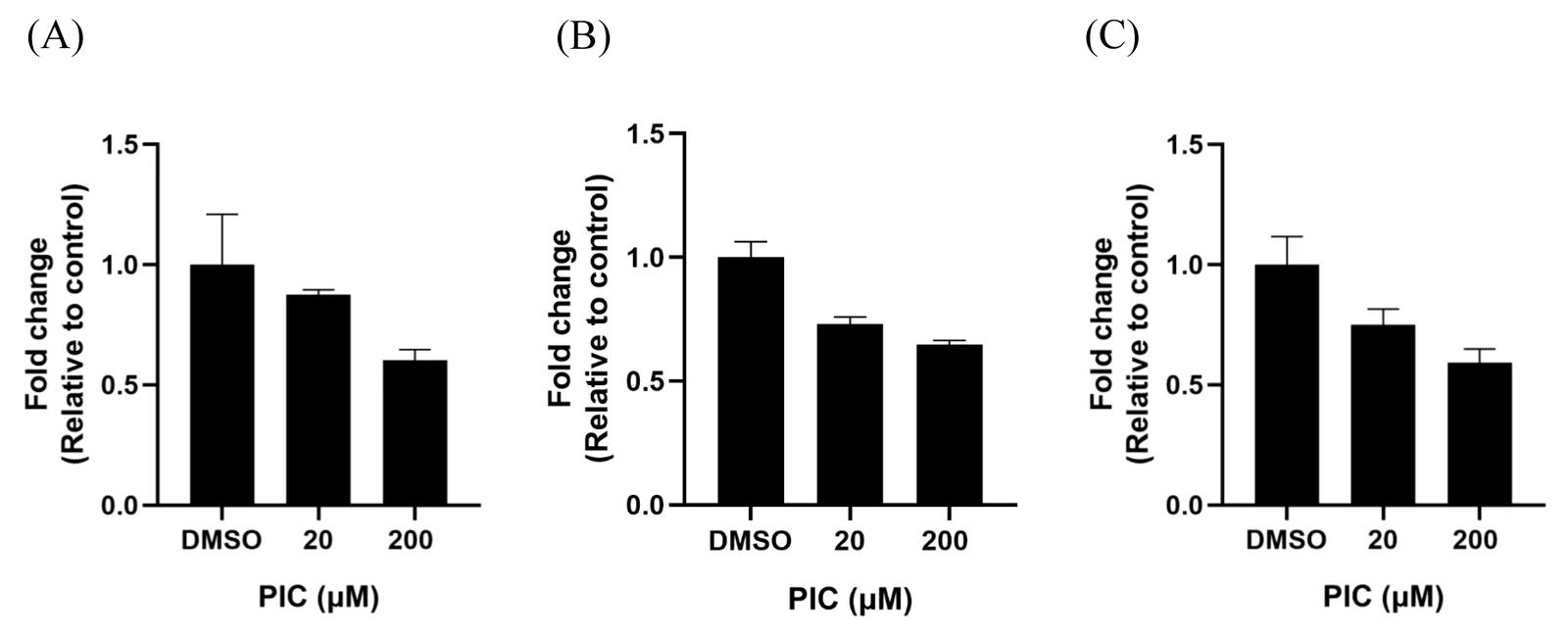
Piceatannol affected the mRNA levels of heat shock protein family D member 1 (HSPD1) in HCC cells. The changes of mRNA levels of HSPD1 after cells treated with various concentrations of piceatannol (PIC) for 48 h were detected using qRT-PCR (quantitative Reverse Transcription-Polymerase Chain Reaction). Assays were performed in Huh7 (A), HepG2 (B) and Hep3B (C) cells. Data were mean ± SD of three technical replicates. No inferential statistical tests were performed.

**Figure 7:**
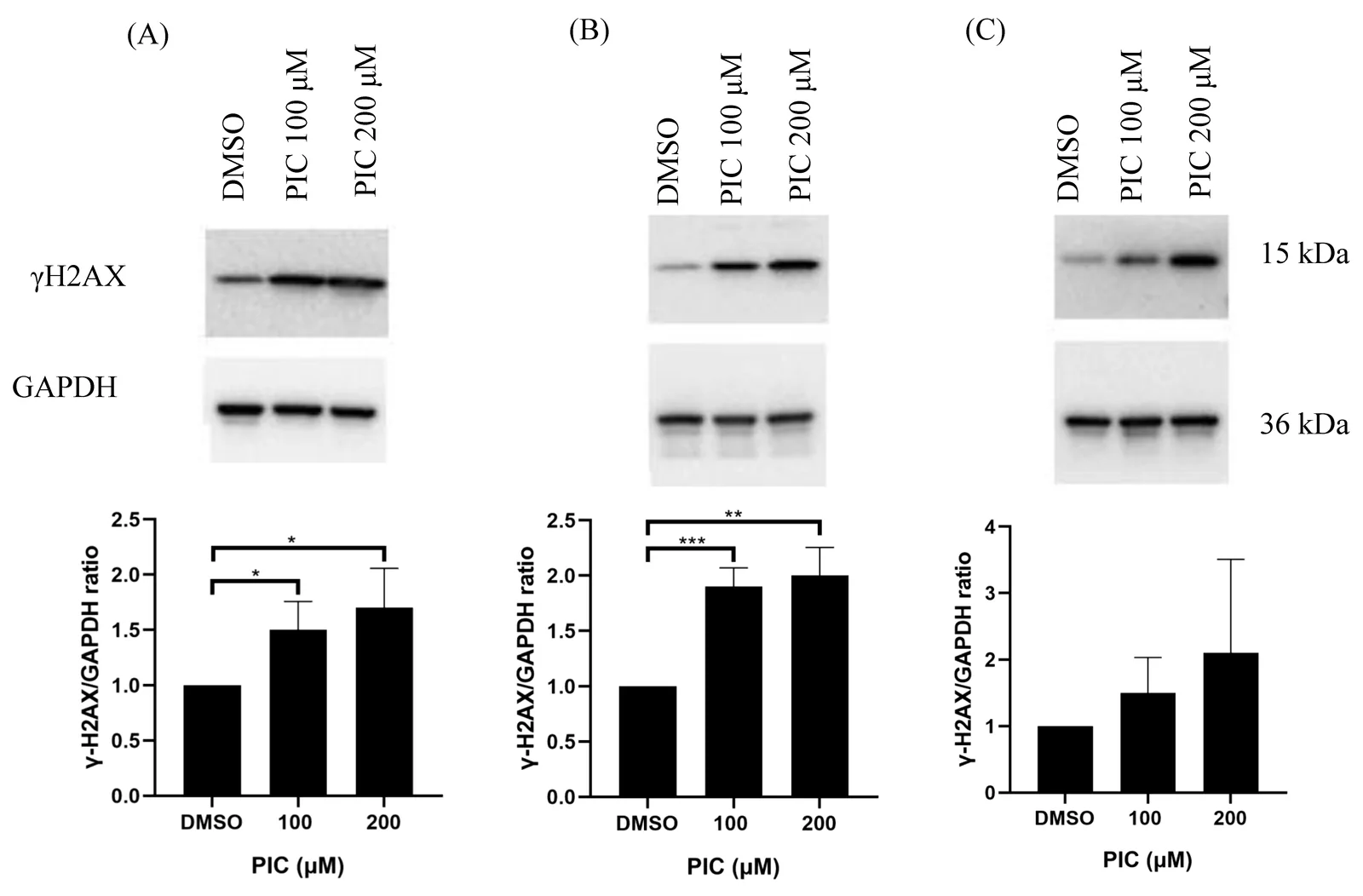
Piceatannol affected the protein expression of γH2AX in HCC cells. Cells were treated with solvent, 100 or 200 μM of piceatannol (PIC) for 48 h and subjected to immunoblot analysis for γH2AX protein expression. GAPDH expression was used as a loading control. Assays were performed in Huh7 (A), HepG2 (B), and Hep3B cells (C) (n=3 biological replicates per group). Statistical significance was determined by one-way ANOVA followed by Dunnett's multiple-comparison test. *p < 0.05, ** p < 0.01, ***p<0.001 versus the control group.

**Figure 8:**
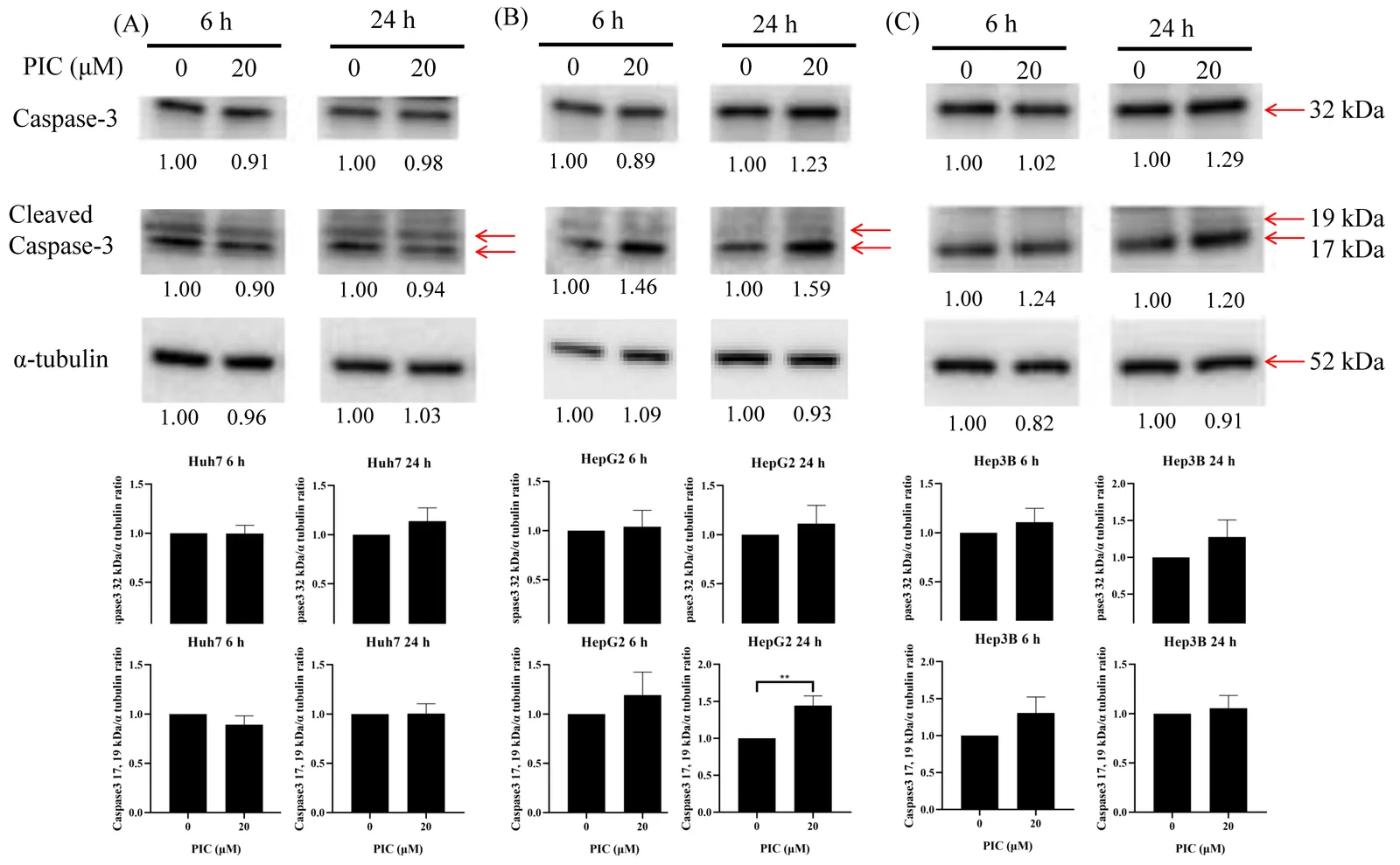
Effects of piceatannol on the expression of total and cleaved Caspase-3 in HCC cells. Huh7 (A), HepG2 (B), and Hep3B (C) cells were treated with 0 or 20 μM of piceatannol (PIC) for 6 or 24 h. Whole cell lysates were collected and subjected to immunoblot analysis for total Caspase-3 and cleaved Caspase-3 protein expression. α-Tubulin expression was used as a loading control. Data were presented as means ± the standard deviations (n=3 biological replicates per group). Statistical significance was determined by one-way ANOVA followed by Dunnett's multiple-comparison test. ** p < 0.01 versus the control group. All values were normalized to the untreated control (0 μM) and expressed as relative fold changes.
