# Supplementary material for: A naturally occurring polyphenolic compound for liver cancer treatment
Source: Int J Med Sci. 2026 Oct 2;23(11):3531–46. doi: 10.7150/ijms.127785 (PMC13685437; doi:10.7150/ijms.127785)
Supplement: Supplementary file 1 [file ijmsv23p3531s1.pdf]

A naturally occurring polyphenolic compound for liver cancer treatment

Ming-Hui Yang[1,+], Yu-Chang Tyan[2,3,4,5,6,+], Hsin-Rou Liang[1,7,+], Chia-Yen Dai[8,9], Yung-Jen Chuang[10], Cheng-Hui Yuan[11], and Shih-Chang Chuang[1,8*]

Cell culture

Human hepatic stellate cells (LX-2) were grown in DMEM (Gibco BRL, Grand Island, NY, USA) enriched with 2% fetal bovine serum (FBS)(HyClone, Logan, UT, USA), penicillin (100 U/mL), streptomycin (100 μg/mL), and L-glutamine (2 millimolar) under a controlled incubator with 5% CO_2.

Western blotting

Cells were treated with 0 or 20 μM piceatannol for 6 h (for Bcl-2 and Bax) or for 24 h (for LC3 & PARP1) before protein extraction. Cell were lysates with radio-immunoprecipitation assay (RIPA) lysis buffer containing 50 mM Tris (pH= 7.5), 150 mM NaCl, 1% Triton X-100, 0.1% sodium dodecyl sulfate (SDS), and 0.5% sodium deoxycholate, This solution was enhanced by protease and phosphatase inhibitor cocktails (04693132001 and 04906837001, Sigma-Aldrich, St. Louis MI, USA). Total protein lysates were quantified using the Bradford assay (Bio-Rad#5000006). Approximately 30 μg total protein was loaded per lane, and equal amounts of proteins were separated by SDS-PAGE before transfer onto polyvinylidene fluoride (PVDF) membrane. Later, the PVDF membranes were blocked with 5% skim milk before incubating overnight at 4 °C with primary antibodies. Then, the membranes were rinsed with PBST and then incubated with diluted secondary antibodies (1:10000) at room temperature for two hours. After rinsing with PBST for a few times, the visualization of the protein band signals were achieved via Enhanced Chemiluminescent Reagent Kit

(ECL) (Visual Protein; LF16-500) using Bio-Rad Western blot imaging systems (Bio-Rad, Hercules, USA). Band quantification was performed using Bio-Rad Image Lab (version 6.1) with α-Tubulin as the loading control. The following antibodies were used: Bcl-2 (1:500, Novus; NB100-56098, Centennial, Co, USA), Bax (1:500, Novus; NB100-56096, Centennial, Co, USA), LC3 (1:1000, Proteintech; 14600-1-AP, Rosemont, IL, USA), PARP1 (1:16000, Proteintech; 13371-1-AP, Rosemont, IL, USA) and α-Tubulin (1:5000, Novus; NB100-690, Centennial, CO, USA).

Hoechst 33342/PI Double Staining

To assess cell viability and mode of cell death, a dual-staining assay with Hoechst 33342 and Propidium Iodide (PI) was performed. Cells were seeded in 12-well plates and treated with piceatannol for the indicated concentrations. Following treatment, the cells were incubated with 2 μg/mL Hoechst 33342 and 2 μg/mL PI in the dark for 10 min at 37°C. After incubation, cells were rinsed twice with PBS to remove excess dye. Stained cells were immediately observed under a Leica DMi8 microscope (Leica Microsystems, Wetzlar Deutschland, Germany) equipped with appropriate filters. Hoechst 33342, which stains all nuclei (blue), and PI, which penetrates only cells with damaged membranes (red), were used to distinguish live, apoptotic, and necrotic cells.

Cell proliferation assay

Cell proliferation was performed to evaluate the effect of piceatannol on cell viabilities and was detected using a MTT proliferation assay. Briefly, approximately 3,000 cells per well were seeded. After the incubation of 24 hours, cells were treated with various doses of piceatannol. MTT solution was added and allowed to incubate for 1.5 hours. Then, DMSO was added to each test well and absorbances were measured using a microplate reader (BioTek Synergy H1, Agilent, Santa Clara, CA, USA) at a

wavelength of 540 nm.

Animal study

A C57BL/6J male mouse was acquired from the National Center for Biomodels (NCB), National Institute of Applied Research (NIAR) in Taiwan. The animal experiment was executed in accordance with the guidelines and regulations for the Care and Use of Laboratory Animals and permitted by the Institutional Animal Care and Use Committee of Kaohsiung Medical University (IACUC #112027). The mouse was kept under a 12-h light–dark cycle room. Briefly, piceatannol was suspended in 0.5% CMC-Na and administered via oral gavage (p.o.) as a single dose of 300 mg/kg body weight. Following administration, body weight and general clinical signs were monitored twice weekly for 14 days. On day 14, the mouse was euthanized for further analysis. The serum sample was obtained and the biochemical data were obtained from a multi-purpose automatic dry-chemistry analyzer (FUJIFILM Dri-Chem NX-500, Ratingen, Germany)

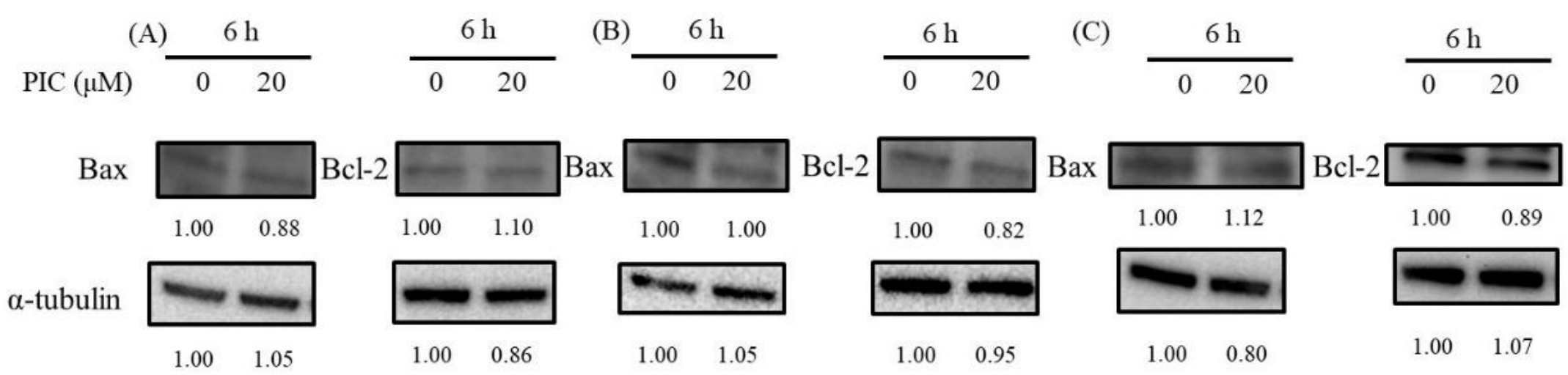

Supplementary Figure S1. Effects of piceatannol on the expression of Bax and Bcl-2 in HCC cells. Huh7 (A), HepG2 (B), and Hep3B (C) cells were treated with 0 or 20 μM of piceatannol (PIC) for 6 hours. Whole cell lysates were collected and subjected to immunoblot analysis of Bax and Bcl-2 protein expression. α-Tubulin expression was used as a loading control. (n=1) All values were normalized to the untreated control (0 μM) and expressed as relative fold changes.

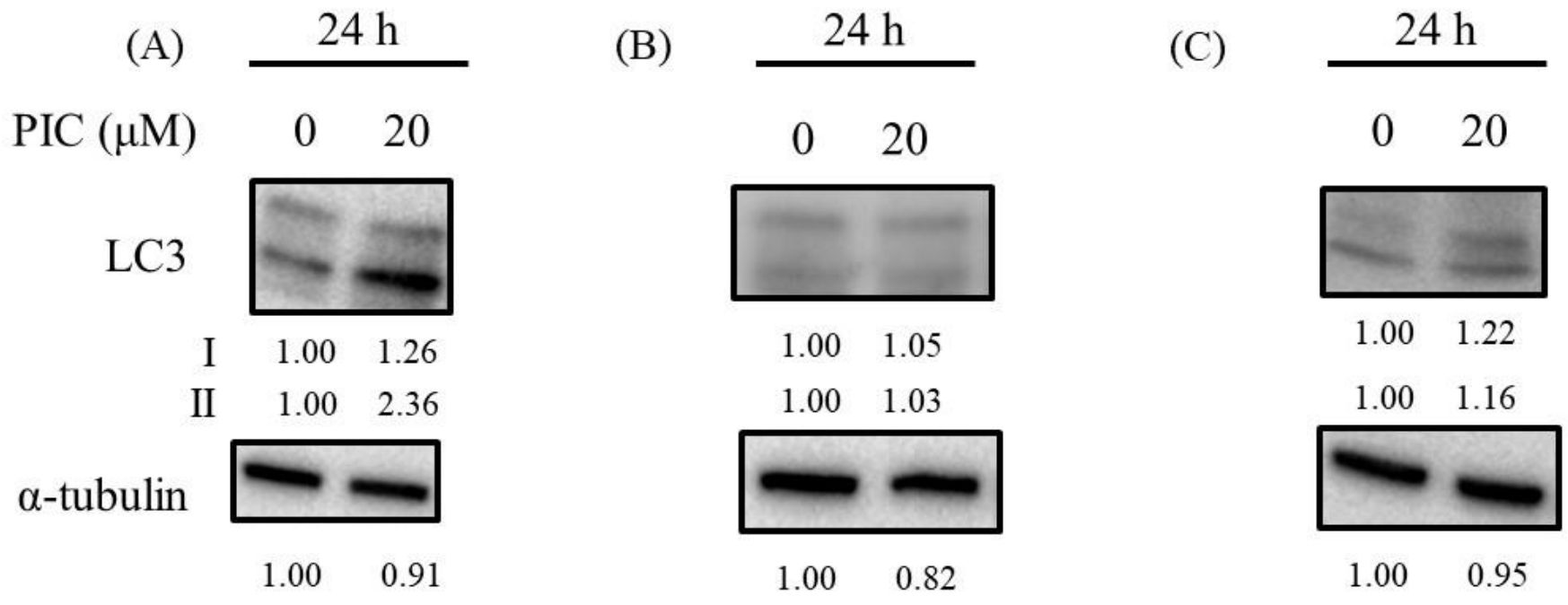

Supplementary Figure S2. Effects of piceatannol on the expression of LC3 in HCC cells. Huh7 (A), HepG2 (B), and Hep3B (C) cells were treated with 0 or 20 μM of piceatannol (PIC) for 24 hours. Whole cell lysates were collected and subjected to immunoblot analysis of LC3protein expression. α-Tubulin expression was used as a loading control. (n=1) All values were normalized to the untreated control (0 μM) and expressed as relative fold changes.

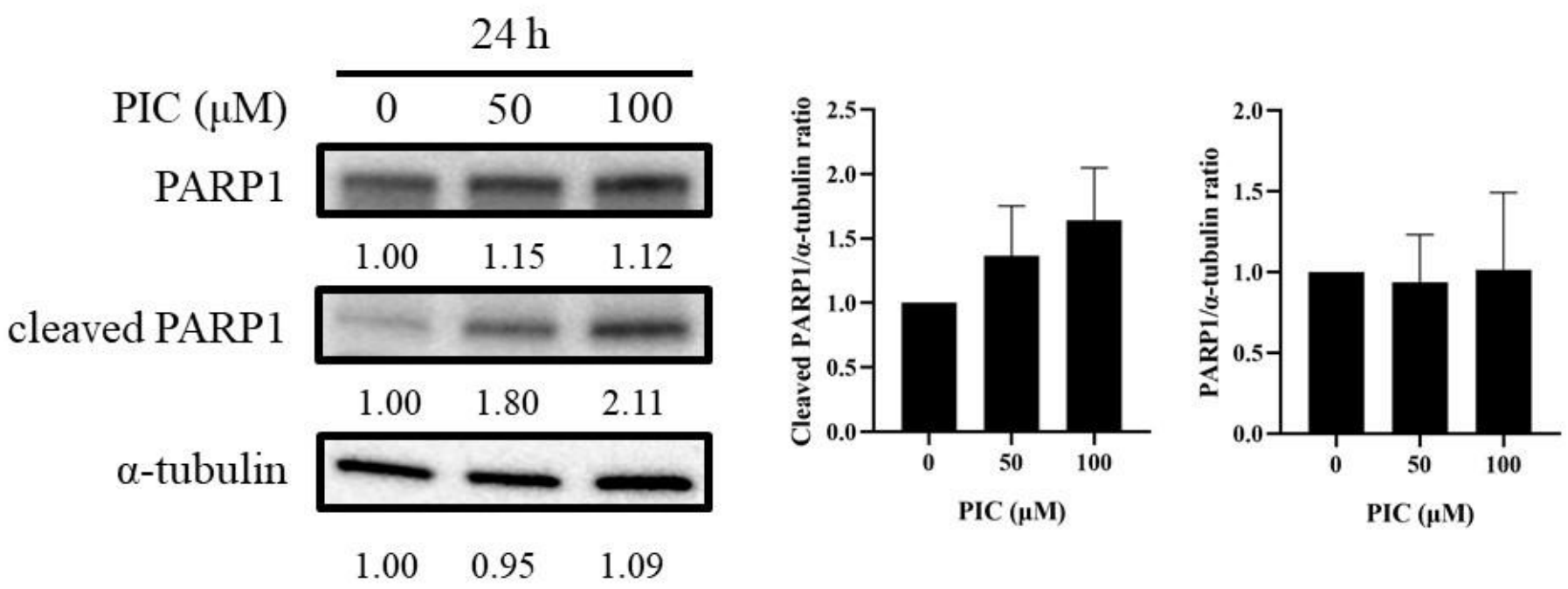

PIC concentration (mM)	0	50	100
Mean of cleaved PARP1/total PARP1 ratio	1.00	1.50	1.91

Supplementary Figure S3. Effects of piceatannol on the expression of PARP1 and cleaved PARP1 in Huh7 cells. Cells were treated with 0, 50 or 100 μM of piceatannol (PIC) for 24 hours. Whole cell lysates were collected and subjected to immunoblot analysis of PARP1 protein expression. α-Tubulin expression was used as a loading control. All values were normalized to the untreated control (0 μM) and expressed as relative fold changes. Results are presented as means ± the standard deviations (n = 3 technical replicates per group)

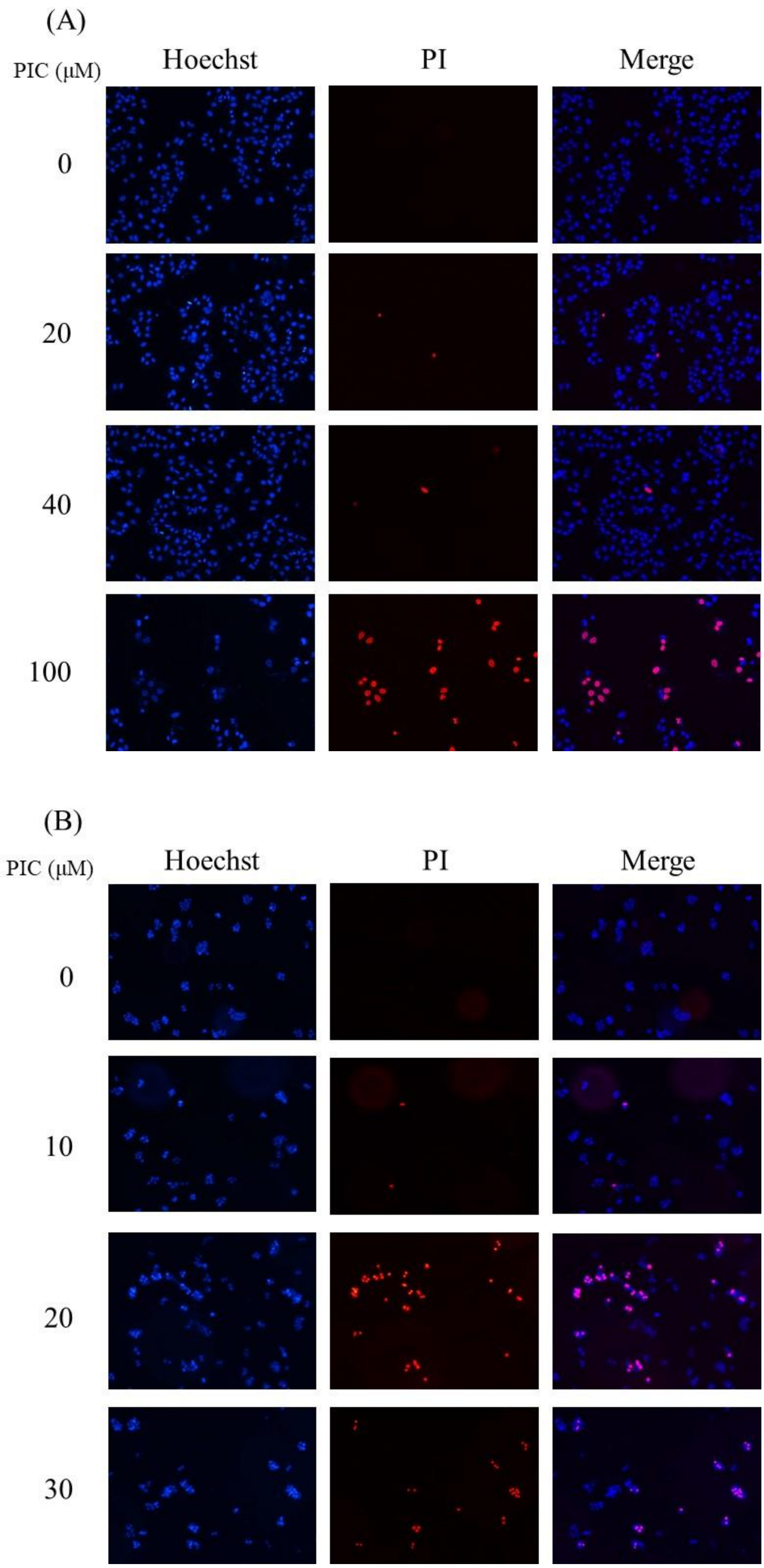
(A)
PIC (μM)
Hoechst
PI
Merge
0
20
40
100
(B)
PIC (μM)
Hoechst
PI
Merge
0
10
20
30

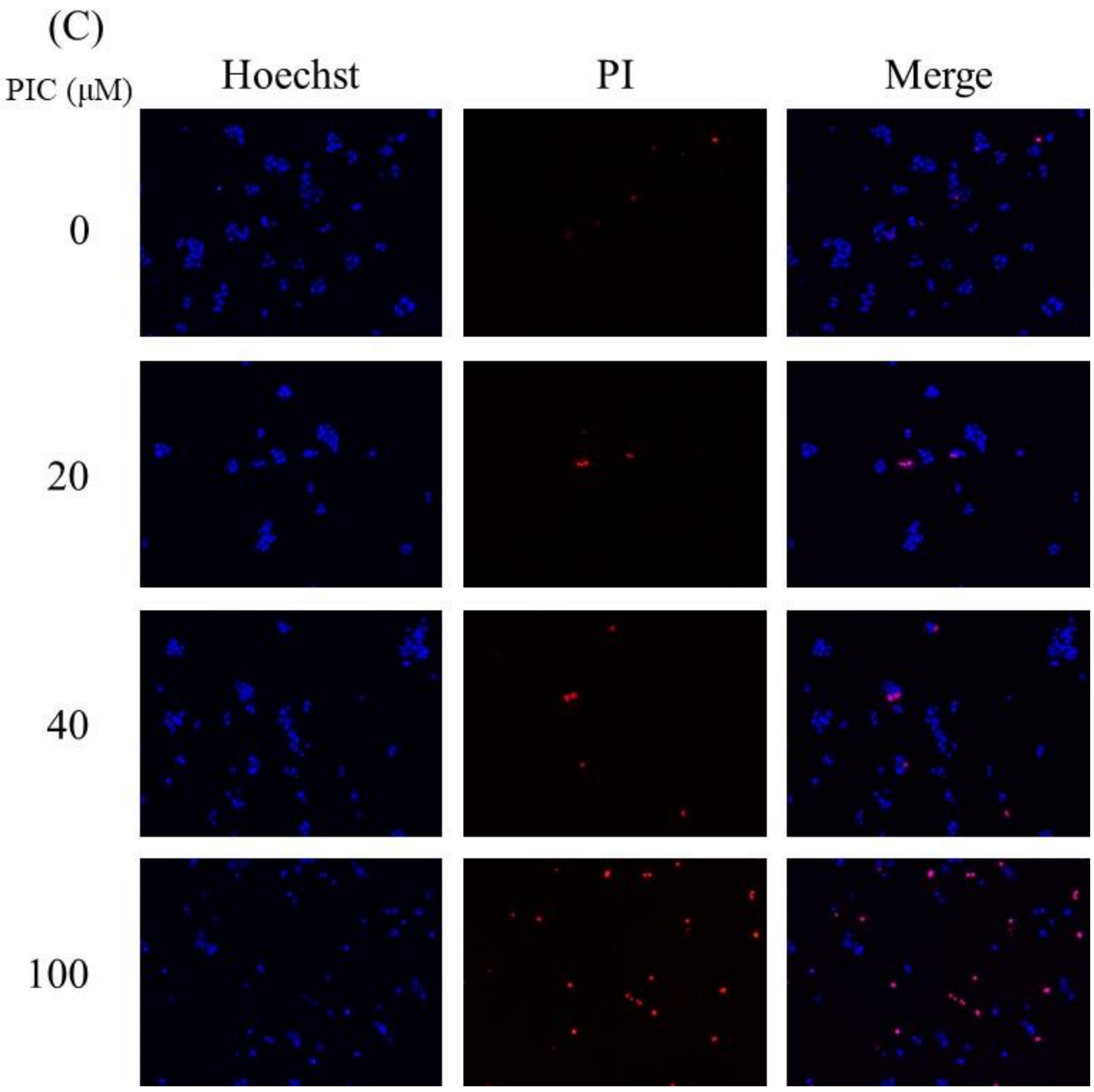

Supplementary Figure S4. Effects of piceatannol on the cell death using Hoechst 33342/Propidium Iodide (PI) double staining. Huh7 (A), HepG2 (B), or Hep3B (C) cells were treated with indicated concentrations of PIC (from top to bottom: control, low, middle, and high concentrations) for 24 hours, followed by dual staining with Hoechst 33342 and propidium iodide (PI) for 10 minutes at 37°C in the dark. Left panel (Blue): Hoechst 33342 staining showing total nuclear morphology. Middle panel (Red): PI staining indicating cells with compromised membrane integrity (dead/late apoptotic cells). Right panel (Merge): Overlaid images demonstrating the co-localization of condensed/fragmented nuclei with PI signals (pink color). Representative images were captured by fluorescence microscopy (Magnification: 100×).

Supplementary Table SI. The cell viability of LX-2 human hepatic stellate cells treated with piceatannol

Concentration (μM)	Cell Viability (%, Mean)	Standard Deviation (SD)
0	100	2.5
3.9	106.8	7.9
7.8	115.6	2.6
15.6	100.9	15.1
31.3	117.8	12.0
62.5	85.3	6.8
125	80.9	6.6
250	63.8	48.0
500	56.4	43.1
1000	58.7	45.4

Supplementary Table SII. The results of CBC tests and serum biochemical tests from a mouse treated with piceatannol.

		Unit	Normal range values
Gender	Male		
Age	11.67	month	
Body weight at initial	31.6	g	
Body weight at sacrifice	28.9	g	
Liver weight	1.2	g	
AST	72	U/L	97.7±19.7
ALT	24	U/L	35.4±13
Alkaline phosphatase	82	U/L	280.6±27
Total protein	4.4	g/dL	5.10±0.54
BUN	22	mg/dL	28.66±4.58
Creatinine	0.16	mg/dL	0.27±0.05
Uric acid	1.0	mg/dL	3.00±0.4
RBC	8.95	million/uL(mm^3)	7.0-12.5×10^6 /mm^3
Hemoglobin	12.6	g/dL	10.2-16.6 mg/dl
WBC	932	/uL(mm^3)	6-15×10^3 /mm^3
Hematocrit	43.4	%	39-49%
M.C.V.	48.5	fL	
M.C.H.	14.1	pg	
M.C.H.C.	29.0	g/dL	
Platelet	1068	thousand/uL	
